# Supports and Barriers to Lifestyle Interventions in Women with Gestational Diabetes Mellitus in Australia: A National Online Survey

**DOI:** 10.3390/nu15030487

**Published:** 2023-01-17

**Authors:** Angelo Sabag, Lauren Houston, Elizabeth P. Neale, Hannah E. Christie, Lauren A. Roach, Joanna Russell, Colin H. Cortie, Marijka Batterham, Barbara J. Meyer, Monique E. Francois

**Affiliations:** 1 Illawarra Health and Medical Research Institute, Wollongong, NSW 2522, Australia; 2 NICM Health Research Institute, Western Sydney University, Westmead, NSW 2145, Australia; 3 The George Institute for Global Health, University of New South Wales, Sydney, NSW 2042, Australia; 4 Faculty of Medicine, University of New South Wales, Sydney, NSW 2052, Australia; 5 School of Medical, Indigenous and Health Sciences, University of Wollongong, NSW 2522, Australia; 6 School of Health and Society, University of Wollongong, Wollongong, NSW 2522, Australia; 7 Graduate School of Medicine, University of Wollongong, Wollongong, NSW 2522, Australia; 8 National Institute for Applied Statistics Research Australia, University of Wollongong, Wollongong, NSW 2522, Australia; 9 Molecular Horizons, University of Wollongong, Wollongong, NSW 2522, Australia

**Keywords:** behaviour change, exercise, physical activity, nutrition, pregnancy, glycaemia, gestational diabetes management

## Abstract

Background: Gestational diabetes mellitus (GDM) affects approximately one in six pregnancies, causing a significant burden on maternal and infant health. Lifestyle interventions are first-line therapies to manage blood glucose levels (BGLs) and prevent future cardiometabolic complications. However, women with GDM experience considerable barriers to lifestyle interventions; thus, the aim of this study was to determine how women with GDM manage their condition and to identify the primary supports and barriers to lifestyle intervention participation. Methods: An online cross-sectional survey of women in Australia with a history of GDM was conducted. Questions included participant demographics, strategies used to manage BGLs, physical activity and dietary habits, and barriers and supports to lifestyle interventions. Results: A total of 665 individuals consented and responded to the advertisement, of which 564 were eligible and provided partial or complete responses to the survey questions. Most respondents were between 35 and 39 years of age (35.5%), not pregnant (75.4%), working part-time (26.7%), university-educated (58.0%), and had only one child (40.1%). Most respondents managed their BGLs through diet (88.3%), with “low-carbohydrate” diets being the most popular (72.3%), and 46.2% of respondents were undertaking insulin therapy. Only 42.2% and 19.8% of respondents reported meeting the aerobic and strengthening exercise recommendations, respectively. Women with one child or currently pregnant expecting their first child were 1.51 times more likely (95% CI, 1.02, 2.25) to meet the aerobic exercise recommendations than those with two or more children. The most common reported barriers to lifestyle intervention participation were “lack of time” (71.4%) and “childcare” commitments (57.7%). Lifestyle interventions delivered between 6 and 12 months postpartum (59.0%), involving an exercise program (82.6%), and delivered one-on-one were the most popular (64.9%). Conclusion: Most women report managing their GDM with lifestyle strategies. The most common strategies reported involve approaches not currently included in the clinical practice guidelines such as reducing carbohydrate consumption. Furthermore, despite being willing to participate in lifestyle interventions, respondents report significant barriers, including lack of time and childcare commitments, whereas mentioned supports included having an online format. Lifestyle interventions for women with a history of GDM should be designed in a manner that is both tailored to the individual and considerate of existing barriers and supports to participation.

## 1. Introduction

Gestational diabetes mellitus (GDM) is a common pregnancy-related complication and is defined as any degree of glucose intolerance with onset or first recognition during pregnancy [1]. In Australia, GDM affects one in six pregnancies, and although only representing 3.6% of all diabetes cases, the incidence of GDM relative to all pregnancies has tripled from 5.2% in 2001 to 16.1% in 2018 [2]. The increased incidence of GDM can cause a significant burden on the health system, and maternal and infant health may become compromised as a result of GDM, which is associated with pregnancy-related complications such as pre-eclampsia [3], delivery of infants large for gestational age [4], and non-elective caesarean delivery [5]. Furthermore, offspring of women with uncontrolled GDM often experience reduced insulin sensitivity and impaired glucose tolerance [6]. As a result, GDM management centres on managing maternal blood glucose levels (BGLs) through lifestyle interventions, including medical nutrition therapy as the primary strategy and exogenous insulin administration should lifestyle interventions alone not suffice [7].

Although primary management of GDM centres on managing symptoms during pregnancy, women with a history of GDM are also at an increased risk of developing early onset cardiovascular disease and type 2 diabetes [8,9,10]. The prevalence of type 2 diabetes is particularly high within the first five years postpartum, with a recent systematic review showing that a history of GDM is associated with a 10-fold increase in the likelihood of developing type 2 diabetes [11]. Although the precise mechanisms underlying this observation are unclear, excess early gestation and postpartum maternal weight are associated with future type 2 diabetes [12,13]. However, of these, postpartum weight has been shown to be a greater predictor of type 2 diabetes than antepartum weight [12]. As there are currently no specific guidelines for postpartum management of GDM, current recommendations involve yearly blood glucose assessments and facilitation of healthy lifestyle behaviours in order to prevent future cardiometabolic disease [14,15].

Lifestyle interventions, including diet and exercise, are cornerstone therapies for the management of metabolic disease [16,17]. Previous findings from the Diabetes Prevention Program have shown that intensive lifestyle therapy involving diet modulation and increased physical activity can prevent progression from GDM to type 2 diabetes in approximately 50% of cases [18]. Although effective, these interventions are time-consuming and consequently may be unfeasible in real-world settings, as many mothers with small children report that a lack of time impacts their ability to adopt and adhere to lifestyle interventions [19]. Similarly, because women are often diagnosed with GDM between 24 and 28 weeks of gestation [20], there is a range of lifestyle changes that need to be made quickly to manage maternal glucose concentrations and reduce associated risks. This urgency and overload of information places significant strain and pressure on women, who often report feeling overwhelmed by the need to master their self-management [21,22].

Given the multitude of competing interests, clinical trials assessing the efficacy of interventions among women with GDM has proven challenging, with low recruitment and high attrition rates a common occurrence [23]. Consequently, the aim of this study was to determine how women with a history of GDM manage their condition. A secondary aim was to determine the supports and barriers to participation in lifestyle interventions.

## 2. Methods

### 2.1. Setting

An online cross-sectional survey was developed and delivered using REDCap, and reporting of survey design and results followed the Checklist for Reporting Results of Internet E-Surveys (CHERRIES) [24].

### 2.2. Participants

Participants were eligible to complete the survey if they self-reported having a previous or current GDM diagnosis, were 18 years or older, and were currently living in Australia.

### 2.3. Survey Design and Development

The survey was designed by a multidisciplinary team of researchers comprising of public health specialists, dietitians, and exercise physiologists and modelled on an existing survey [25] (Supplementary File S1). The questionnaire was piloted in a small sample of women with a previous diagnosis of GDM to check for clarity and performance. Demographics of the women surveyed including age, ethnicity, state/territory, marital and employment status, education, pregnancy, and parity were collected, followed by a combination of multiple-choice and open-ended free-text questions to explore women’s practices used to manage their condition, adherence to physical activity and dietary recommendations, and supports and barriers to participation in lifestyle interventions. Adaptive questioning was used, whereby questions were conditionally displayed depending on the response given to previous questions. In addition, respondents were able to review and change their answers as they completed the survey. The survey was anonymous, and there was no direct contact between the respondents and researchers. Answers to all items were voluntary. Respondents were provided with the option to skip or leave question items blank or to choose non-responses, such as “prefer not to say” or “I don’t know”. This study was approved by the University of Wollongong Human Research Ethics Committee (number 2021/346).

### 2.4. Survey Administration

The survey was administered, and data were stored on REDCap (Research Electronic Data Capture), a secure, web-based application designed to support data capture for research studies [26]. Participants responded to targeted social media (Facebook and Instagram) advertisements and were redirected to the survey webpage via an embedded link or QR code. The survey was open between November and December 2021 and from February to March 2022. Informed consent was provided by all women.

### 2.5. Data Analysis

All responses, including those that were partially completed, were included for analysis. Data were analysed using Microsoft Excel and IBM SPSS Statistics (V27, IBM, Armonk, NY, USA). Descriptive statistics were reported as means and standard deviations or percentages. Between-group comparisons were undertaken using the chi-square test, and logistic regression was used to calculate the odds of those with ≤one child meeting the activity guidelines compared with those with ≥2 children. Significance was set at *p* < 0.05. Missing data were not replaced.

## 3. Results

Descriptive data are summarised in Supplementary Table S1. Briefly, 665 individuals consented and responded to the advertisement, of which 564 were eligible and provided partial or complete responses to the survey questions. Those deemed ineligible were either not women or did not have a current or previous diagnosis of GDM. The majority of respondents were between the ages of 30 and 34 (*n* = 194, 34.4%) or 35 and 39 (*n* = 200, 35.5%), self-reported to be non-Aboriginal and/or Torres Strait Islander peoples (*n* = 454, 80.5%), were from Western Australia (*n* = 168, 29.9%), were married (*n* = 366, 65.0%), were working part-time (*n* = 147, 26.7%), had a bachelor’s degree (*n* = 235, 42.0%), were not pregnant (*n* = 410, 75.4%), and had only one child (*n* = 217, 40.1%). Of the participants who were not pregnant at the time of completing this survey, the majority had a body mass index of 30 kg/m^2^ or higher (*n* = 225, 53.2%). Of the participants who were pregnant at the time of completing the survey, the majority had a body mass index of 30 kg/m^2^ or higher (*n* = 74, 53.7%).

**Figure 1:**
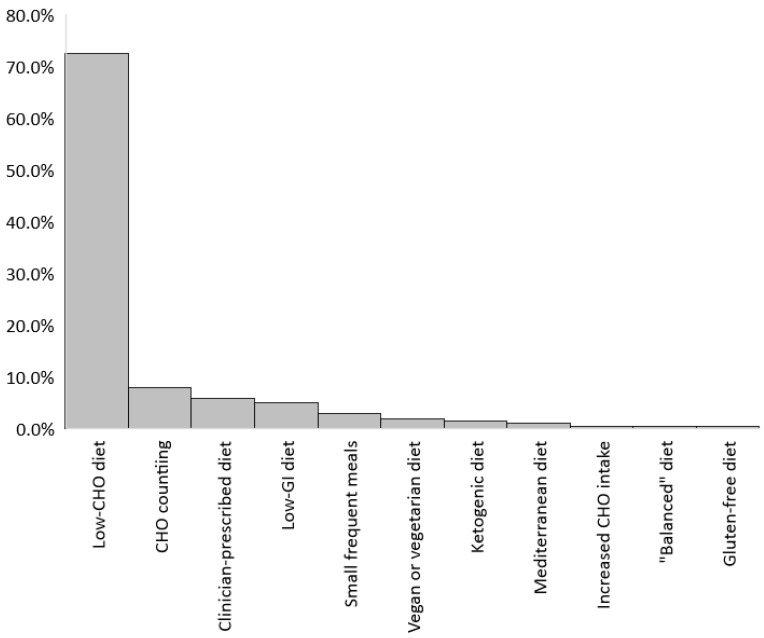
Specific diets followed during pregnancy (*n* = 202).

Of the 202 respondents who reported following a specific diet during pregnancy, the most commonly reported was a low-carbohydrate diet (72.3%), followed by carbohydrate counting (7.9%) (Figure 1).

### 3.1. Strategies for Managing GDM

A total of 293 (55.0%) of 533 respondents reported feeling comfortable receiving advice relating to their pregnancy and GDM from a medical practitioner, 287 (53.8%) from a dietitian, 211 (39.6%) from a midwife, 26 (4.9%) from a diabetes educator, 24 (4.5%) from other sources of information, 10 (1.9%) from a naturopath, and 4 from a doula (0.8%).

A total of 548 respondents provided responses as to how they managed their GDM and the strategies they found effective in managing their blood sugar levels (Table 1). The most common strategies used to manage GDM were following “a healthy eating pattern” (88.3%) and self-monitoring BGLs (88.0%). The strategy most women reported to be effective in managing their BGLs was ‘lowering the amount of carbohydrates in meals and snacks’.

### 3.2. Achieving Physical Activity and Dietary Recommendations

A total of 405 respondents reported on whether they met the aerobic and strengthening exercise recommendations. Less than half of all participants reported meeting the aerobic exercise recommendations (*n* = 171, 42.2%), and less than a quarter of all participants reported meeting the strengthening exercise recommendations (*n* = 80, 19.8%) (Table 2) [27,28]. Of the 340 respondents who met neither the aerobic nor strengthening exercise recommendations, 73.8% indicated that they were willing or very willing to make lifestyle changes to meet the recommendations; however, of that group, only 44.4% believed that they were likely or very likely to realistically meet the recommendations.

A total of 387 participants reported complying with at least one of the five healthy eating strategies derived from Australian dietary guidelines [29] and derived resources (Table 2). A total of 256 respondents who did not meet all of the dietary recommendations were subsequently prompted to indicate whether they would be willing to meet these requirements. Of these, 77.3% indicated that they were willing or very willing to make lifestyle changes to meet the recommendations, but only 64.3% believed that they were likely or very likely to realistically meet the recommendations.

The respondents’ age at the time of completing the survey was not associated with meeting current physical activity guidelines (Table 3). Younger respondents were more willing to participate in a lifestyle intervention (61.3% vs. 50.6% for <35 years and ≥35 years, respectively); however, the discrepancy did not reach statistical significance (*p* = 0.052) Respondents with a tertiary education degree were significantly more likely to be willing to participate in a university-led lifestyle intervention research study (*p* < 0.01) and to report complying with the strengthening exercise recommendations (*p* = 0.02) (Table 3). Women with one child or currently pregnant expecting their first child were 1.51 times more likely (95% CI, 1.02,2.25) to report complying with the current aerobic exercise recommendation than those with two or more children (*p* = 0.04). There was no difference in physical activity behaviours or willingness to participate in lifestyle interventions between women with pregravid obesity (body mass index ≥30 kg/m^2^) versus women without pregravid obesity (body mass index <30 kg/m^2^) (*p* > 0.05 for analyses).

### 3.3. Supports and Barriers to Participation in a Lifestyle Intervention

A total of 470 participants reported that they were unlikely/very unlikely (20.6%), neutral (24.3%), or likely/very likely (55.1%) to participate in a lifestyle intervention run by university researchers. The 373 respondents who reported being neutral, likely, or very likely to participate in a lifestyle intervention were prompted to indicate how and when they would like to participate in a lifestyle intervention program. The respondents preferred lifestyle interventions that began between 6 and 12 months postpartum (59.0%), involved an exercise program (82.6%), and were delivered via one-on-one coaching (64.9%). The respondents also reported that they would prefer to engage in sessions that were delivered fortnightly (49.3%) and online (64.6%). A total of 437 respondents identified barriers to participating in a healthy lifestyle program. The most common barrier to participation was a lack of time (71.4%), followed by childcare (57.7%), work commitments (43.7%), and competing priorities (36.6%) (Table 4).

## 4. Discussion

This study is the first national Australian online survey to explore the management strategies currently used, as well as barriers and supports to lifestyle intervention participation, among women with current or previous GDM. The vast majority of women reported managing their GDM by changing eating patterns and regularly monitoring their BGLs. The most common dietary approach used was a lower-carbohydrate diet. The results of this study also show that most respondents were likely or very likely to be willing to participate in a healthy lifestyle program. Interventions delivered between 6 and 12 months postpartum, involving individual coaching, conducted online, and including exercise programs were identified as the most desirable. The most frequently reported barriers to participation in lifestyle programs were lack of time and childcare commitments. Furthermore, university-educated women were significantly more likely to report meeting the current strengthening exercise recommendations and to be willing to participate in a lifestyle intervention than those without a university education. Women with two or more children were significantly more likely to report not meeting the current aerobic exercise recommendations, suggesting a compounding effect of childcare responsibilities on physical inactivity.

### 4.1. Previous Findings

The findings of this study are similar to those of the United Kingdom (UK) study from which our survey was modelled [25]. For example, the top three intervention programs selected by respondents across the two studies were identical, with the most popular intervention being exercise programs, followed by recipe ideas and dietary advice/counselling. Furthermore, 58% of respondents in this study identified childcare requirements as a significant barrier to lifestyle intervention adoption, which was similar to those from the UK study (64%). However, the results between the studies differed in terms of how respondents preferred to receive the intervention. Most respondents in this study preferred to receive interventions delivered via an online format, whereas respondents in the UK study preferred group sessions. This may have been impacted by the timing of the survey completion, which was conducted during coronavirus disease 2019 (COVID-19) pandemic.

### 4.2. Management Strategies for GDM

The American Diabetes Association recommends that lifestyle interventions should be used as a first-line therapy for the management of hyperglycaemia during pregnancy [7]. In the event that lifestyle intervention alone is not sufficient for improving glycaemia, insulin should be added as a first-line treatment strategy [7]. The results of this study indicate that current treatment practices among women with GDM reflect the guidelines, as the majority of participants reported self-monitoring their BGLs and using diet and physical activity to manage their glucose levels. However, there was an unexpectedly large proportion of respondents who reported taking insulin therapy to manage their GDM compared to the 2019 National Diabetes Service Scheme (NDSS) data (33.8% vs. 46.2% for NDSS data and study respondents, respectively) [30]. Although the reason for such a discrepancy remains unclear, it is possible that participants would have been more willing to undertake lifestyle interventions, and thus complete the survey, in an attempt to avoid future reliance on pharmacotherapy.

The American Diabetes Association recommends medical nutrition therapy for GDM be individualised and developed in conjunction with a registered dietitian familiar with the management of GDM [7,31]. Although there are no specific dietary recommendations for women with GDM, it is recommended that all pregnant women consume a minimum of 175 g of carbohydrates per day (approximately 35% of an 8360 kJ diet [7]), which is reported to be necessary to account for glucose utilisation by the foetus and brain [32]. Interestingly, of the respondents who reported following a specific diet to manage their BGLs, the vast majority of respondents indicated that they had reduced the amount of carbohydrates in meals and snacks. This may be because of the strong emphasis on self-monitoring fasting and postprandial blood glucose in the attempt to keep within tight target ranges. Carbohydrates, which break down into glucose during metabolism, are the primary dietary nutrient that affects postprandial blood glucose responses. Further research exploring the degree to which women with GDM reduce their carbohydrate intake would be valuable. Despite preliminary data supporting the use of lower-carbohydrate diets for the management of blood glucose in GDM, the efficacy of such a strategy has not been fully established [33]. Because most respondents reported seeking advice from their medical practitioner, it may be that clinicians are increasingly advocating for lower-carbohydrate diets despite a lack of clear guidelines, highlighting a key area for future research.

The current Australian guidelines for physical activity and exercise during pregnancy state that women should undertake physical activity on most but preferably all days of the week with the aim of accumulating 150–300 min of moderate-intensity physical activity or 75–150 min of vigorous intensity physical activity or an equivalent combination of both moderate and vigorous activities each week [28]. Additionally, muscle-strengthening activities are recommended on at least 2 days each week [28]. These guidelines are the same as what is recommended for the general adult population [27]. Although half of all respondents reported using physical activity to help manage their GDM, less than half of respondents reported meeting the aerobic exercise recommendations, and less than one-quarter of respondents reported meeting the muscle-strengthening exercise recommendations. Interestingly, many women reported that physical activity/walking after meals was an effective strategy for managing blood glucose. Although only a handful of small acute studies are available to support this, available studies have shown promising results with respect to the glucose-lowering benefits of performing one postprandial bout of exercise [34,35,36] or, alternatively, three shorter bouts of walking after main meals [37]. Although the results of this study include data from both pregnant and non-pregnant women, they highlight that the majority of women with current or previous GDM are not meeting the physical activity recommendations. As adequate levels of physical activity and cardiorespiratory fitness are integral components of cardiometabolic health [38], future studies should aim to explore strategies that improve these indices among women with current or previous GDM.

### 4.3. Barriers and Supports to Lifestyle Interventions

A recent systematic review showed that “lack of time” remains a significant barrier to lifestyle change and maintenance in women with GDM [19]. The results of the present study extend previous findings by once again showing that “lack of time” remains the primary barrier to adopting a healthy lifestyle program, followed by childcare and work commitments. These findings may explain, in part, why women with two or more children were significantly less likely to achieve the aerobic exercise recommendations. Furthermore, respondents willing to participate in a lifestyle program preferred online formats, followed by home visits or outdoor settings for program delivery, which may reduce the childcare barrier. However, a recent ten-week live online webinar program involving group education and exercise for women less than one year postpartum still reported poor adherence (<20%) [39]. Therefore, more flexibility in timing (asynchronous) and/or format and incorporating one-on-one coaching may be more beneficial for future program success. Interestingly, having a tertiary level education or higher was associated with a greater willingness to participate in a lifestyle intervention and to report meeting the current strengthening exercise recommendations. Future interventions could be designed to address this discrepancy by clearly demonstrating the value of lifestyle interventions and strengthening exercises for health.

As previously mentioned, it is well-known that establishing the efficacy of novel lifestyle interventions for GDM is difficult due to low recruitment and high attrition rates in studies involving this population [23]. As the majority of participants were most comfortable receiving management advice from their medical practitioner, future studies should aim to engage and include clinicians in order to increase recruitment numbers and retain participants. Furthermore, the results of this study indicate that most women would prefer to undertake an intervention 6 months postpartum. Although this would not have an impact on their previous pregnancy, implementing an intervention at this time point might elicit improvements in cardiometabolic health outcomes, which can reduce the risk of recurring GDM or ensuing type 2 diabetes. Similarly, the respondents indicated that they are most receptive to receiving an exercise program delivered on a one-on-one basis or via mobile/internet coaching. Consequently, future studies should aim to explore the efficacy of online exercise interventions that have the capacity to be scaled and implemented in the community.

### 4.4. Limitations

This study has several limitations that should be considered when interpreting the results. First, this study, by nature, was conducted at a single time point, and as a result, it is not possible to determine the change in habits, barriers, or supports to lifestyle interventions in women with GDM. Secondly, this study was designed to include women with both current and previous GDM. As a result, it is unclear whether being pregnant or recalling behaviours following a GDM pregnancy would affect the responses. Thirdly, this study was conducted during the COVID-19 pandemic, for which many daily activities were migrated to online formats. Consequently, the proportion of women who reported being willing to undertake an online intervention may have differed had the questionnaire been deployed prior to the pandemic. Additionally, this study primarily recruited individuals with access to online social media platforms, which may have also affected the results. Fourthly, this study was conducted in Australia, and as a result, the findings may differ relative to those of studies conducted in other countries, particularly in countries with limited access to appropriate pre- and perinatal healthcare. Fifthly, GDM can present with many phenotypes, and as a result, the findings presented in this study are relevant to GDM per se but may be less applicable to specific GDM phenotypes, such as those with normal pregravid BMI. As a result, future research is needed to identify the barriers and supports to lifestyle interventions in different GDM phenotypes. Finally, many participants, either by questionnaire design or by personal choice, did not answer all questions. Consequently, there is a large discrepancy in the number of responses across questions. However, where possible, the total number of respondents and associated percentage was indicated in the Results section.

## 5. Conclusions

The results of this national survey among women with current or previous GDM show that lifestyle interventions are sufficient to manage BGLs in most GDM cases, as less than half of all respondents reported using insulin to manage their BGLs. Furthermore, the results of this study also suggest that although lifestyle interventions are highly desired, key barriers such as lack of time and childcare commitments must be mitigated to increase participation. Future studies assessing the efficacy of lifestyle interventions in GDM may have greater recruitment and retention rates if they are delivered between 6 and 12 months postpartum and involve one-on-one or online exercise programs, as these were reported to be the most desirable intervention characteristics.

## Figures and Tables

**Table 1:** Strategies for managing GDM.

**How Do/Did You Manage Your Gestational Diabetes?**	**Insulin Therapy** ***n*** ** = 253 (%)**	**Non-Insulin Therapy *n* = 295 (%)**	**Total** ***N* = 548 (%)**	***p* Value **
A healthy eating pattern	219 (86.6%)	265 (89.8%)	484 (88.3%)	0.235
Self-monitoring blood glucose levels	222 (87.7%)	260 (88.1%)	482 (88.0%)	0.889
Physical activity	120 (47.4%)	163 (55.3%)	283 (51.6%)	0.068
Metformin	39 (15.4%)	32 (10.8%)	71 (13.0%)	0.112
I do not know	0 (0%)	3 (1%)	3 (0.5%)	0.108
Other	0 (0%)	3 (1%)	3 (0.5%)	0.108
**Have You Found Any of the Following Strategies Effective in Managing Your Blood Glucose Levels?**	**Insulin Therapy** ***n*** ** = 253 (%)**	**Non-Insulin Therapy** ***n*** ** = 295 (%)**	**Total** ***N* = 548 (%)**	***p* Value **
Lowering the amount of carbohydrates in meals/snacks	222 (87.7%)	191 (64.7%)	413 (75.4%)	0.600
Walking/physical activity after meals	105 (41.5%)	133 (45.1%)	238 (43.4%)	0.506
Consuming a supper/evening snack	108 (42.7%)	117 (39.7%)	225 (41.1%)	0.351
Other	16 (6.3%)	16 (5.4%)	34 (6.2%)	0.624

**Table 2:** Responses to meeting the physical activity and diet recommendations for gestational diabetes mellitus.

**Currently Meeting Physical Activity Recommendations**	***n***	**%**
Aerobic exercise recommendations	171	42.2
Strengthening exercise recommendations	80	19.8
Respondents	405	
**Currently Undertaking Healthy Eating Practices**	***n***	**%**
Eat small amounts often and maintain a healthy weight	172	44.4
Include some carbohydrates in every meal and snack (e.g., multigrain bread, bulgur, pasta, potato, lentils, chickpeas, beans)	317	81.9
Choose a wide variety of nutritious foods	331	85.5
Avoid foods and drinks containing large amounts of sugar	269	69.5
Choose foods that have a low glycaemic index and will help you to stay fuller for longer	236	64.3
Respondents	387	

**Table 3:** Compliance with physical activity guidelines stratified by education and number of children.

	**<35 Years**	**≥35 Years**	**Total**	***p* Value**
Currently meeting aerobic exercise guidelines				
Yes	83 (43.2%)	88 (41.3%)	171 (42.2%)	0.697
No	109 (56.8%)	125 (58.7%)	234 (57.8%)	
Total (*n*)	192	213	405	
Currently meeting strengthening exercise guidelines				
Yes	35 (18.2%)	45 (21.1%)	80 (19.8%)	0.465
No	157 (81.8%)	168 (78.9%)	325 (80.2%)	
Total (*n*)	192	213	405	
Likely to participate in lifestyle intervention trial				
Likely	144 (61.3%)	115 (50.6%)	259 (56.0%)	0.052
Neutral	48 (20.4%)	66 (29.1%)	114 (24.7%)	
Unlikely	43 (18.3%)	46 (20.3%)	89 (19.3%)	
Total (*n*)	235	227	462	
	**Tertiary Education**	**No Tertiary Education**	**Total**	***p* Value**
Currently meeting aerobic exercise guidelines				
Yes	109 (44.5%)	61 (38.9%)	170 (42.3%)	0.264
No	136 (55.5%)	96 (61.1%)	232 (57.7%)	
Total (*n*)	245	157	402	
Currently meeting strengthening exercise guidelines				
Yes	58 (23.7%)	22 (14.0%)	80 (19.9%)	**0.018**
No	187 (76.3%)	135 (86.0%)	322 (80.1%)	
Total (*n*)	245	157	402	
Likely to participate in lifestyle intervention trial				
Likely	170 (61.8%)	86 (44.8%)	256 (54.8%)	**<0.001**
Neutral	58 (21.1%)	56 (29.2%)	114 (24.4%)	
Unlikely	47 (17.1%)	50 (26.0%)	97 (20.8%)	
Total (*n*)	275	192	467	
	**Two or More Children**	**One Child or Less**	**Total**	***p* Value**
Currently meeting aerobic exercise guidelines				
Yes	78 (37.3%)	91 (47.4%)	169 (42.1%)	**0.041**
No	131 (62.7%)	101 (52.6%)	232 (57.9%)	
Total (*n*)	209	192	401	
Currently meeting strengthening exercise guidelines				
Yes	41 (19.6%)	39 (20.3%)	80 (20.0%)	0.862
No	168 (80.4%)	153 (79.7%)	321 (80.0%)	
Total (*n*)	209	192	401	
Likely to participate in lifestyle intervention trial				
Likely	126 (51.4%)	130 (59.4%)	256 (55.2%)	0.165
Neutral	67 (27.3%)	45 (20.5%)	112 (24.1%)	
Unlikely	52 (21.2%)	44 (20.1%)	96 (20.7%)	
Total (*n*)	245	219	464	

*p* values derived from chi-squared test.

**Table 4:** Supports and barriers to lifestyle intervention participation.

**When would you be willing to participate in a healthy lifestyle intervention? ** **(*n* = 373)**	***n***	**%**
Pre-pregnancy	147	39.4
Trimester 1	126	33.8
Trimester 2	144	38.6
Trimester 3	141	37.8
<6 months postpartum	131	35.1
6–12 months postpartum	220	59.0
>12 months postpartum	207	55.5
Never	13	3.5
**What would you be willing to participate in?** **(*n* = 373)**	***n***	**%**
Exercise program	308	82.6
Recipe ideas	275	73.7
Dietary counselling	273	73.2
Wellbeing counselling	246	66
Cooking session	158	42.4
None	13	3.5
Other	2	0.5
**What is your preferred choice of delivery to receive information?** **(*n* = 373)**	***n***	**%**
Individual coaching (1:1)	242	64.9
Mobile/Internet coaching	234	62.7
External coach (e.g., Fitbit or mobile application)	204	54.7
Group coaching	112	30
Telephone	109	29.2
None	12	3.2
Other	4	1.1
**How would you like to engage in session(s)?** **(*n* = 373)**	***n***	**%**
Online	241	64.6
Home visits	144	38.6
Community hall	72	19.3
University research setting	74	19.8
Medical setting (for example, doctor’s consultation room)	142	38.1
Outdoor setting (for example, parks or a playground)	165	44.2
None	4	1.1
Other	3	0.8
**How often would you like to attend/receive your choice of delivery? ** **(*n* = 373)**	***n***	**%**
Once a week	170	45.6
Once a fortnight (every 2 weeks)	184	49.3
Once a month (every 4 weeks)	102	27.3
Once every 2 months (ever 8 weeks)	27	7.2
Once every 3 months (every 12 weeks)	21	5.6
Unsure	35	9.4
Never	1	0.3
**Barriers to lifestyle intervention** **(*n* = 437)**	***n***	**%**
Lack of time	312	71.4
Childcare	252	57.7
Work commitment	191	43.7
Competing priorities	160	36.6
Finances	124	28.4
Family support	105	24
Social support	30	6.9
Physical environment	26	5.9

## Data Availability

The datasets generated and/or analysed for the current study are available from the corresponding author upon reasonable request.

## Supplementary Materials

The following are available online at https://www.mdpi.com/article/10.3390/nu15030487/s1, File S1: Questionnaire, Table S1: Respondent demographic, employment, and educational characteristics.

## Abbreviations

COVID-19	Coronavirus disease 2019
GDM	Gestational diabetes mellitus
BGL	Blood glucose level
CHERRIES	Checklist for Reporting Results of Internet E-Surveys
NDSS	National Diabetes Service Scheme
UK	United Kingdom
