# Supplementary material for: Supports and Barriers to Lifestyle Interventions in Women with Gestational Diabetes Mellitus in Australia: A National Online Survey
Source: Nutrients. 2023 Jan 17;15(3):487. doi: 10.3390/nu15030487 (PMC9921280; doi:10.3390/nu15030487)
Supplement: Supplementary file 1 [file nutrients-15-00487-s001.zip › nutrients-2096005-supplementary File S1.pdf]

Welcome to our Nutrition and Physical Activity Survey for Women with or have had prior Gestational Diabetes. This survey will help inform our advice and future programs to support women for better health and wellbeing during this time.You are invited to take part in this online survey. Please read this information carefully. Ask questions about anything that you don’t understand or want to know more about.

I have been provided with the Participant Information Sheet (attached below) about this survey. I have been advised of the potential risks and burdens associated with this research, which include completing a 10 to 15 minute online survey.

I understand that my participation in this research is voluntary, I am free to refuse to participate and I am free to withdraw from the research at any time. If I choose not to participate or withdraw consent it will not affect my treatment in anyway and/or my relationship with the University of Wollongong.

If I have any enquiries about the research, I can contact Dr Monique Francois (Email: francois@uow.edu.au) who is a researcher at the University of Wollongong. If I have any concerns or complaints regarding the way the research is or has been conducted, I can contact the Ethics Officer, Human Research Ethics Committee, Office of Research, University of Wollongong on Ph. (02) 42213386 or Email: mailto:rso-ethics@uow.edu.au

There’s also a chance to win one of two $50 VISA gift cards. Each survey submission is anonymous – contact details for the prize draw are collected at the end of the survey in a way that protects the anonymity of feedback provided. The details on how to enter the prize draw are provided at the end of the survey.

By agreeing to the below I am indicating my consent to participate in this online survey. Please select the following statements if they apply to you (select all that apply):

◯ I consent to be in this study
◯ I do NOT consent to be in this study - this will end the survey

---

Please select the following statements if they apply to you (select all that apply): (select all that apply):

☐ I am female
☐ I am currently or have been previously diagnosed with gestational diabetes

About you

Please follow instructions and select the most appropriate response(s) from the range of options or type free text in the box provided for the following questions.

What is your age? (Select one)

◯ Under 20 years old
◯ 20 - 24 years old
◯ 25 - 29 years old
◯ 30 - 34 years old
◯ 35 - 39 years old
◯ 40 years or older

With which ethnic group do you identify? (Select all that apply)

☐ Australian
☐ Indigenous Australian or Torres Strait Islander
☐ New Zealand
☐ Asian
☐ Indian
☐ African
☐ European
☐ Middle eastern
☐ North American
☐ South American
☐ Decline to answer
☐ Other

Please specify:

____

Where is your home located?

◯ Victoria
◯ Tasmania
◯ Queensland
◯ South Australia
◯ Western Australia
◯ New South Wales
◯ Northern Territory
◯ Australian Capital Territory
◯ Prefer to not say
◯ Other

Please specify:

____

What is your postcode?

____

What is your marital status? (Select one)

◯ De facto
◯ Divorced
◯ Married
◯ Never married
◯ Separated
◯ Widowed
◯ Prefer to not say

What is your current employment status? (Please select all that apply)

☐ Full-time
☐ Part time
☐ Casually
☐ Full-time carer
☐ Currently not working
☐ Currently on maternity leave
☐ Prefer to not say
☐ Other

Please specify:

____

What is your highest level of completed education? (Select one)

◯ Did not complete high school
◯ High school
◯ College/TAFE course (e.g. apprenticeship)
◯ Bachelor degree (including Honours)
◯ Masters degree (including course work and research degrees)
◯ Doctoral degree

The following set of questions are about your current or past pregnancy(s) and diagnosis with gestational diabetes.

Are you currently pregnant? (Select one)

◯ Yes
◯ No
◯ Prefer not to say

How many weeks pregnant are you?

____
(For example, if you are 25 weeks write 25.)

How long has it been since you were last diagnosed with gestational diabetes? (Select one)

◯ Less than 1 year
◯ 2 to 4 years
◯ 5 to 7 years
◯ More than 8 years
◯ Prefer not to say

How many children are you caring for excluding your current pregnancy? (Select one)

◯ 0
◯ 1
◯ 2
◯ 3
◯ 4 or more
◯ Prefer not to say

How many children are you caring for? (Select one)

◯ 0
◯ 1
◯ 2
◯ 3
◯ 4 or more
◯ Prefer not to say

How many pregnancies have you been diagnosed with gestational diabetes? (Select one)

◯ 1
◯ 2
◯ 3
◯ 4
◯ More than 4
◯ Prefer not to say

Are you planning a future pregnancy? (Select one)

◯ Yes
◯ No
◯ Unsure
◯ Prefer not to say

How do you manage your gestational diabetes? (Select all that apply)

☐ Physical activity
☐ A healthy eating pattern
☐ Self-monitoring blood glucose levels
☐ Insulin injections
☐ Metformin
☐ I do not know
☐ Other

Please specify:

____

How did you manage your gestational diabetes? (Select all that apply)

☐ Physical activity
☐ A healthy eating pattern
☐ Self-monitoring blood glucose levels
☐ Insulin injections
☐ Metformin
☐ I do not know
☐ Other

Please specify:

____

Have you found any of the following strategies effective in managing your blood sugar? (Select all that apply)

☐ Consuming a supper/evening snack
☐ Lowering the amount of carbohydrates in meals/snacks
☐ Walking/physical activity after meals
☐ Other

Please specify:

____

Did you find any of the following strategies effective in managing your blood sugar? (Select all that apply)

☐ Consuming a supper/evening snack
☐ Lowering the amount of carbohydrates in meals/snacks
☐ Walking/physical activity after meals
☐ Other

Please specify:

____

Regarding your current pregnancy and gestational diabetes diagnosis, who do you feel most comfortable receiving advice from? (Select all that apply)

☐ Dietitian
☐ Doula
☐ Medical doctor
☐ Midwife
☐ Naturopath
☐ Other

Please specify:

____

Regarding your previous pregnancy(s) with gestational diabetes, who did you feel most comfortable receiving advice from?

☐ Dietitian
☐ Doula
☐ Medical doctor
☐ Midwife
☐ Naturopath
☐ Other

Please specify:

____

What is your height in centimetres (cm)?

____
(For example, if you are 1.72 meters (m) or 172 centimetres, (cm) write 172.)

What was your pre-pregnancy weight in kilograms (kg)?

____

What is your current weight in kilograms (kg)?

____

The following questions are related to your likeliness to participate in a research study that involves a healthy lifestyle program.

How likely are you to participate in a research study that involves a healthy lifestyle program (including weight management, diet, physical activity and wellbeing) run by university researchers? (Select one)

◯ Very unlikely
◯ Unlikely
◯ Neutral
◯ Likely
◯ Very likely

Please explain:

At what time would you be willing to participate in a healthy lifestyle program? (Select all that apply)

☐ Pre pregnancy
☐ Early pregnancy (trimester 1)
☐ Mild pregnancy (trimester 2)
☐ Late pregnancy (trimester 3)
☐ Less than 6 months post pregnancy
☐ Between 6 to 12 months post pregnancy
☐ More than 12 months post pregnancy
☐ Never

Would you be willing to participate in: (Select all that apply)

☐ Recipe ideas
☐ Cooking session
☐ Dietary counselling
☐ Exercise program
☐ Wellbeing counselling
☐ None
☐ Other

Please specify:

____

What is your preferred choice of delivery to receive information? (Select all that apply)

☐ Group coaching
☐ Individual (1:1) coaching
☐ Telephone coaching
☐ Mobile/internet coaching
☐ External coaching (for example, Fitbit or mobile application)
☐ None
☐ Other

Please specify:

____

How would you like to engage in session(s)? (Select all that apply)

- ☐ Online
- ☐ Home visits
- ☐ Community hall
- ☐ University research setting
- ☐ Medical setting (for example: doctors consultation room)
- ☐ Outdoor setting (for example: parks or a playground)
- ☐ None
- ☐ Other

Please specify:

____

How often would you like to attend/receive your choice of delivery? (Select all that apply)

- ☐ Once a week
- ☐ Once a fortnight (every 2 weeks)
- ☐ Once a month (every 4 weeks)
- ☐ Once every 2 months (ever 8 weeks)
- ☐ Once every 3 months (every 12 weeks)
- ☐ Unsure
- ☐ Never

Do any of the following impact your ability to participate in a research study that involves a healthy lifestyle program (including weight management, diet, physical activity and wellbeing)? (select all that apply)

- ☐ Childcare
- ☐ Social support
- ☐ Family support
- ☐ Finance support
- ☐ Time availability
- ☐ Paid employment
- ☐ Physical environment
- ☐ Competing priorities
- ☐ Lack of knowledge around how to manage a healthy lifestyle
- ☐ Other

Please specify:

____

How likely are you to discuss with a research team your... (Select 'Very unlikely', 'Unlikely', 'Neutral', 'Likely' or 'Very likely' for each row below)?

	Very unlikely	Unlikely	Neutral	Likely	Very likely
current or previous gestational diabetes diagnosis	○	○	○	○	○
eating patterns	○	○	○	○	○
physical activity	○	○	○	○	○
weight	○	○	○	○	○
wellbeing (overall physical, social and emotional health)	○	○	○	○	○
mental health (feelings of stress, anxiety, depression)	○	○	○	○	○

What would make you feel more comfortable to discuss this with a research team?

Do you meet the current recommendations for physical activity described below? (Select 'Yes' or 'No' for each row below)

	Yes	No
2.5 - 5 hours of moderate - vigorous aerobic exercise each week (i.e., walking, cycling, dancing, swimming)	◯	◯
With strengthening activities on at least 2 days each week (yoga, pilates or weights etc)	◯	◯

Would you be willing to make any lifestyle changes to meet these physical activity recommendations?

◯ Very unlikely
◯ Unlikely
◯ Neutral
◯ Likely
◯ Very likely

Realistically, do you see yourself meeting this guideline?

◯ Very unlikely
◯ Unlikely
◯ Neutral
◯ Likely
◯ Very likely

Do you currently meet the dietary recommendations as described below? (Select 'Yes' or 'No' for each row below)

We should follow a healthy eating plan and are encouraged to:

	Yes	No
Eat small amounts often and maintain a healthy weight	◯	◯
Include some carbohydrate in every meal and snack (e.g. Multigrain bread, bulgur, pasta, potato, lentils, chickpeas, beans)	◯	◯
Choose a wide variety of nutritious foods	◯	◯
Avoid foods and drinks containing large amounts of sugar	◯	◯
Choose foods that have a lower glycaemic index and will help you to stay fuller for longer.	◯	◯

Would you be willing to make any lifestyle changes to meet these dietary recommendations?

◯ Very unlikely
◯ Unlikely
◯ Neutral
◯ Likely
◯ Very likely

Realistically, do you see yourself meeting these recommendations?

◯ Very unlikely
◯ Unlikely
◯ Neutral
◯ Likely
◯ Very likely

Do you currently follow a special diet or eating pattern? (For example: low carbohydrate, ketogenetic (very low carbohydrate) diet, intermittent fasting or Mediterranean diet).

◯ Yes
◯ No
◯ Don't know
◯ Prefer not to say

Please specify:

____

Regarding your previous pregnancy(s) with gestational diabetes, did you follow a special diet or eating pattern? (For example: low carbohydrate, ketogenetic diet, intermittent fasting or Mediterranean diet).

◯ Yes
◯ No
◯ Don't know
◯ Prefer not to say

Please specify:

____
