# Supplementary material for: Supports and Barriers to Lifestyle Interventions in Women with Gestational Diabetes Mellitus in Australia: A National Online Survey
Source: Nutrients. 2023 Jan 17;15(3):487. doi: 10.3390/nu15030487 (PMC9921280; doi:10.3390/nu15030487)
Supplement: Supplementary file 1 [file nutrients-15-00487-s001.zip › nutrients-2096005-supplementary Table S1.pdf]

Table S1. Respondent demographic, employment, and educational characteristics.

Age (Years)	<20	20–24	25–29	30–34	35–39	>40			Total
Number	0	12	63	194	200	95			564
%	0	2.1	11.2	34.4	35.5	16.8			
Pregravid BMI*	<25 kg/m^2	25–29.9 kg/m^2	30–34.9 kg/m^2	35–39.9 kg/m^2	>40 kg/m^2				
Number	29	35	31	28	15				138
%	21.0	25.4	22.5	20.3	10.9				
Current non-pregnant BMI†	<25 kg/m^2	25–29.9 kg/m^2	30–34.9 kg/m^2	35–39.9 kg/m^2	>40 kg/m^2				
Number	82	116	123	49	53				423
%	19.4	27.4	29.1	11.6	12.5				
State	VIC	QLD	SA	WA	NSW	NT	ACT	PNA	Total
Number	24	30	112	168	161	1	64	2	562
%	4.3	5.3	19.9	29.9	28.6	0.2	11.4	0.4	
Marital Status	De facto	Divorced	Married	NM	Separated	Widowed	PNA		Total
Number	142	10	366	13	21	1	10		563
%	25.5	1.8	65.0	2.3	3.7	0.2	1.8		
Employment	FT	PT	Casual	FT carer	Not working	ML	PNA	Other	Total
Number	146	147	23	17	71	125	11	11	551
%	26.5	26.7	4.2	3.1	12.9	22.7	2.0	2.0	
Education	<HS	HS	TAFE/ College	Bachelor's degree	Master's degree	Doctoral Degree			Total
Number	19	64	152	235	74	16			560
%	3.4	11.4	27.1	42.0	13.2	2.9			
Time since GDM diagnosis	<1 year	2–4 years	5–7 years	> 8 years					Total
Number	164	173	39	34					410
%	40.0	40.2	9.5	8.3					
Children	0	1	2	3	≥4				Total
Number	46	217	189	63	26				541
%	8.5	40.1	34.9	11.6	4.8				

* Data for women who were pregnant at the time of completing the survey. † Data for women who were not pregnant at time of completing survey. BMI, body mass index; VIC, Victoria; QLD, Queensland; SA, South Australia; WA, Western Australia; NSW, New South Wales; NT, Northern Territory; ACT, Australian Capital Territory; PNA, prefer not to answer; NM, not married; FT, full-time; PT, part-time; ML, maternity leave; HS, high school; GDM, gestational diabetes mellitus.
